# Fibula-related complications during bilateral tibial lengthening: 60 patients followed for mean 5 years

**DOI:** 10.3109/17453674.2012.665328

**Published:** 2012-06-04

**Authors:** Seung-Ju Kim, Mandar Vikas Agashe, Sang-Heon Song, Hae-Ryong Song

**Affiliations:** Korea University Medical Center, Guro Hospital, Seoul, South Korea

## Abstract

**Background and purpose:**

Complications related to the fibula during distraction osteogenesis could cause malalignment. Most published studies have analyzed only migration of the fibula during lengthening, with few studies examining the effects of fibular complications.

**Patients and methods:**

We retrospectively reviewed 120 segments (in 60 patients) between 2002 and 2009. All patients underwent bilateral tibial lengthening of more than 5 cm. The mean follow-up time was 4.9 (2.5–6.9) years.

**Results:**

The average lengthening percentage was 34% (21–65). The ratio of mean fibular length to tibial length was 1.05 (0.91–1.11) preoperatively and 0.83 (0.65–0.95) postoperatively. The mean proximal fibular migration (PFM) was 15 (4–31) mm and mean distal fibular migration (DFM) was 9.7 (0–24) mm. Premature consolidation occurred in 10 segments, nonunion occurred in 12, and angulation of fibula occurred in 8 segments after lengthening. Valgus deformities of the knee occurred in 10 segments.

**Interpretation:**

PFM induced valgus deformity of the knee, and premature consolidation of the fibula was associated with the distal migration of the proximal fibula. These mechanical malalignments could sometimes be serious enough to warrant surgical correction. Thus, during lengthening repeated radiographic examinations of the fibula are necessary to avoid complications.

Extensive lower limb lengthening often introduces complications including genu varum, genu valgum, tibia vara, ankle varus, and ankle valgus which may require another surgical correction (Barreto et al. 2007).

Soft tissue-resisting forces during the lengthening of bone are responsible for most of the complications. The resisting forces can be soft tissues, interosseus membrane, or bone itself in tibial distraction (Saleh et al. 2002, Hatzokos et al. 2004, Shyam et al. 2009). These resisting forces produce a drag effect on the fibula and hence there is decreased distraction, which is responsible for complications such as tibial axial deviation, and distraction at the proximal and distal tibiofibular joints. Several studies have found migration of the proximal and distal fibula during lengthening (Saleh et al. 2002, Hatzokos et al. 2004) but there is no clinical research regarding long-term outcome of the complications of the fibula itself such as premature consolidation, nonunion, and angulation. In an earlier study (Shyam et al. 2009), we reported increased knee laxity and valgus angulation of the tibia due to proximal fibular migration. In the present study of 120 lengthened tibial segments, we have investigated whether the various complications of the fibula affected the axial limb alignment and had a bearing on the joint-related complications.

## Patients and methods

We retrospectively studied 60 patients (35 men, 120 tibial segments) all of whom underwent bilateral tibial lengthening with Ilizarov ring fixator at our institute between 2002 and 2009. The average age of the patients at the time of surgery was 16 (8–25) years. The etiology was achondroplasia in 32 patients, hypochondroplasia in 15, idiopathic short stature in 10, spondyloepiphyseal dysplasia in 2, and spondylometaphyseal dysplasia in 1 patient. Average follow-up of patients was 4.9 (2.5–6.9) years.

The Ilizarov ring fixator (U & I, Seoul, Korea) was used for the tibial lengthening. 3 or 4 rings were used, depending on whether unifocal or bifocal osteotomies were done at the center of rotation of angulation (CORA) for gradual lengthening. Paired hinges were aligned with the apex of deformity and the lengthening rod was placed opposite them. We used 2 Ilizarov wires each at the proximal and distal ring. Proximal and distal tibiofibular joints were each transfixed with an Ilizarov wire to prevent distal or proximal migration of the fibula. We inserted 2 additional tibial half-pins each at the proximal and middle ring. The osteotomy using multiple drill-hole method was done at the level of the CORA.

Lengthening with or without gradual correction of deformity was started after 7 days at a rate of 1 mm/day (0.25 mm every 6 h). The rate was adjusted during follow-ups according to callus morphology in radiographs. Removal of the fixator was done when 3 cortices showed satisfactory corticalization on radiographs. A long leg cast was applied after fixator removal, and was removed after 4–6 weeks. Postoperative radiographic measurements were based on immediate post-removal radiographs. All radiographs were taken using StarPACS (INFINITT, Seoul, Korea) and PiView STAR software version 5.0.6.0 with X-ray beams perpendicular to the center of the distraction site.

Patients had postoperative follow-up at 2, 4, 6, and 8 weeks and then every month thereafter for 1 year. During visits, they were examined for any signs of pin tract infection, range of motion of adjacent joints, angulation or translation of the osteotomy site, and other complications that could occur during lengthening.

The radiographs were evaluated with regard to 7 parameters: (1) mechanical axis deviation (MAD), measured on long standing radiographs preoperatively and at the last follow-up; (2) preoperative and postoperative (final follow-up) fibula to tibia ratio (FTR), calculated by dividing the length of the fibula by the length of the tibia; (3) tibiofibular distraction difference (TFDD), calculated by subtracting total fibular distraction from total tibial distraction at the end of the distraction period; (4) proximal fibular migration (PFM), calculated by measuring the distal migration of the fibular head by comparing the positions of the fibular head relative to the tibial plateau on the preoperative radiograph and the radiograph made at the end of the distraction period; (5) distal fibular migration (DFM), calculated by measuring the proximal migration of the lateral malleolus by comparing the positions of the lateral malleolus relative to the medial malleolus on the preoperative radiograph and the radiograph taken at the end of the consolidation period; and (6) tibial alignment, calculated by comparing the orientation of the joint lines of the knee and ankle on preoperative radiographs with those on the postoperative radiographs. Regarding knee deformity, a value of >10 mm valgus MAD was assumed to be clinically important because a residual deformity of such magnitude might warrant surgical correction (Aldegheri 1999). The mean external fixation index (EFI) was computed by dividing the number of days in the external fixator by the final regenerate length (Isaac et al. 2008).

### Statistics

The data were analyzed using SAS 9.2 software. The results of the amount of lengthening and the other parameters are denoted as mean (minimum–maximum) values. The 2 bone segments of each patient were considered as separate random units and as such were analyzed separately using the linear mixed-effects model. This model was fitted with the assumption of the unstructured co-variance matrix, considering the fact that the unstructured matrix is the most “liberal” matrix—allowing every item of the analysis to be different. The individual subjects (patients) were considered to be categorical covariates into which various fixed and random effects were observed. The sides (right/left) and groups (according to lengthening percentage) were denoted as fixed effects while other factors such as PFM, DFM, and MAD were considered as random effects. In cases of 3 or more effects, the test was first performed overall and if the effect was statistically significant, then multiple comparisons were performed between the various groups. Any p-value < 0.05 was considered to be statistically significant.

## Results

### Lengthening and other indices

The average gain in length was 9.1 (6.8–12.2) cm and the mean lengthening percentage (LP) was 34% (21–65). The mean external fixator index (EFI) was 28 (11–80) days/cm. The mean mechanical axis deviation (MAD) was 16 (1–29) mm preoperatively and 8.7 (20 of varus to 20 of valgus) mm at the final follow-up. The mean fibula to tibia ratio (FTR) was 1.05 (0.91–1.11) preoperatively and 0.83 (0.65–0.95) postoperatively. The mean tibiofibular distraction difference (TFDD) was 23.8 (5–51) mm, and the mean proximal fibular migration (PFM) and mean distal fibular migration (DFM) were 15.2 (4–31) mm and 9.7 (0–24) mm, respectively.

### Complications

**Figure 1.**
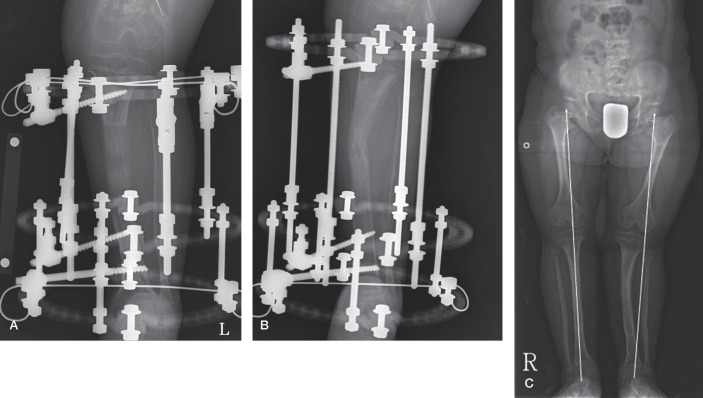
A. A 10-year-old girl with achondroplasia. Premature consolidation of the fibula osteotomy site and distal migration of the proximal fibula with cutting out of the proximal wire from the fibula occurred. B. Varus angulation of the tibia at 6 months. C. Valgus deformity of the left knee and distal migration of the proximal fibula, 4 years after surgery.

**Figure 2.**
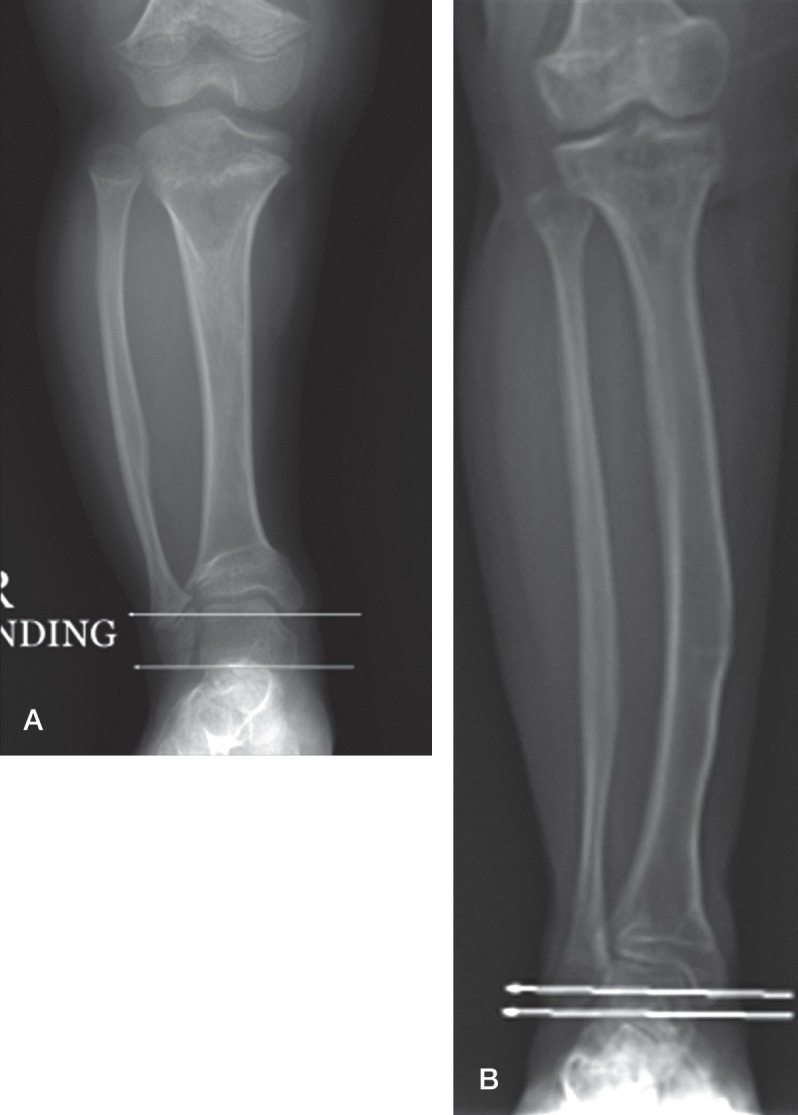
A. An 11-year-old girl with achondroplasia. Preoperative standing. The proximal line shows the level of the tip of the medial malleolus while the distal line denotes the level of the tip of the lateral malleolus. B. Proximal migration of the distal fibula with valgus deformity of the right ankle. Proximal migration of the distal fibula was measured by comparing the amount of the lateral malleolus projecting distal to the medial malleolus on preoperative and postoperative radiographs.

Fibula complications occurred in 30 of the 120 segments during lengthening. There were 10 segments with premature consolidation, 12 segments with nonunion, and 8 segments with angulation of the fibula. All patients showed PFM (Figure 1) and 96 segments showed DFM (Figure 2). Valgus deformity of the knee occurred in 10 segments, and the mean MAD of these 10 segments was 15 (12–20) mm of valgus. All patients with valgus deformity of the knee showed PFM of more than 10 mm. The mean angulation of the fibula was 5.1 (1.5–16) degrees at the final follow-up. Cut-out of the wire from the fibula occurred in 10 segments. There was only 1 segment with cut-out of the wire in patients who had lengthening of less than 25% and 9 segments with cut-out of the wire were in patients who had lengthening of more than 25%. Equinus contracture was the commonest complication with 20 segments. The mean equinus deformity was 14 (5–19) degrees after surgery and 4.2 (0–9) degrees after removal of the external fixator. Recession of gastrocnemius aponeurosis to correct equinus deformity was performed.

**Figure 3.**
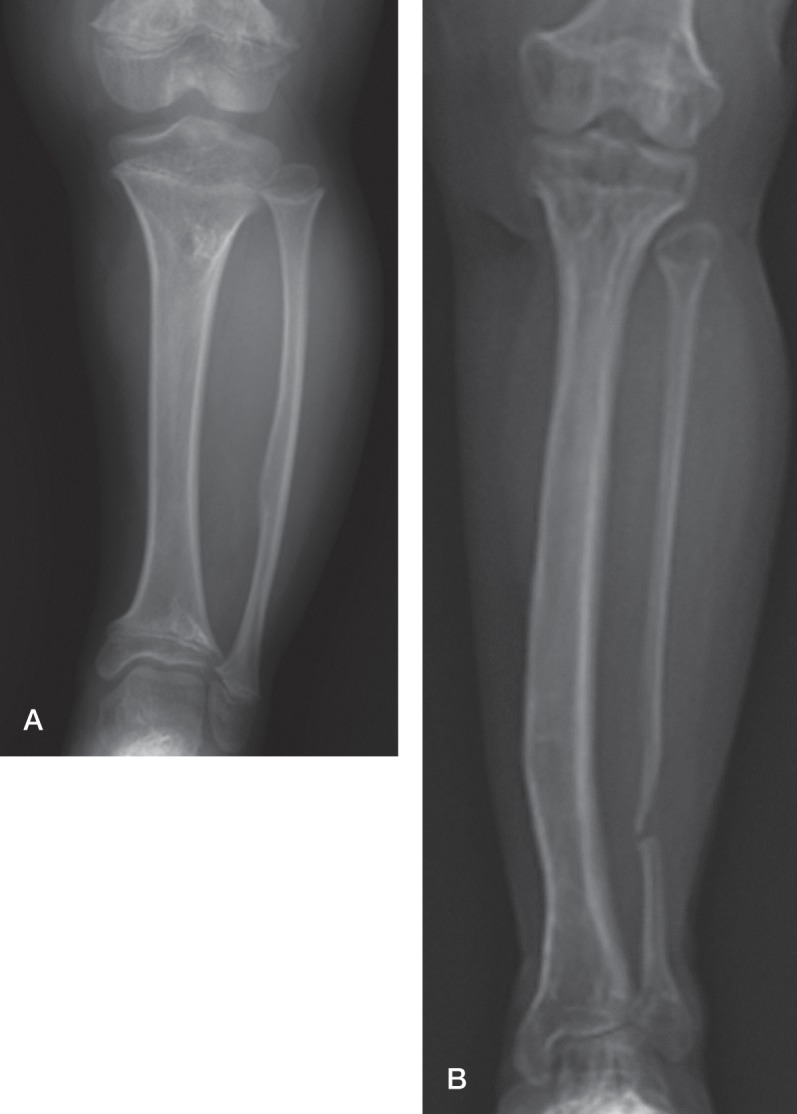
A. An 11-year-old girl with achondroplasia. Preoperative standing. B shows nonunion of fibula osteotomy site with valgus deformity of the ankle, 2 years after surgery.

Valgus of the knee (valgus change of MAD) increased with increased lengthening. PFM and the amount of lengthening were interrelated to some extent, even though it was not statistically significant (p = 0.08). PFM was associated with valgus deformity of the knee (p = 0.03). PFM and valgus change of MAD showed statistically significant differences depending on lengthening percentage (Table 1). Premature consolidation of the fibula resulted in complications around the knee with a significant increase in PFM, while nonunion of the fibula was associated with complications around the ankle with a significant increase in the DFM (Table 2 and Figure 3).

**Table 1.** Comparison of various parameters according to lengthening group. Analysis was performed using the linear mixed-effects model. Mean values are given

Lengthening percentage	No. of segments	Amount of lengthening (cm)	PFM **^a^** (mm)	DFM **^b^** (mm)	MAD **^c^** (mm)
< 25	11	5.7	17.9 **^d, e^**	8.9 **^d, e^**	1.2
25–50	61	8.5	19.5 **^d^**	6.6 **^d^**	3.0
> 50	48	10.8	13.4 **^e^**	7.6 **^e^**	2.7
p-value			0.002	0.1	< 0.001

**^a ^**PFM: proximal fibula migration

**^b ^**DFM: distal fibula migration

**^c ^**MAD: mechanical axis deviation

**^d, e^** Same letters indicate no statistical significance, based on a mixed-effects model.

**Table 2.** Statistical results of comparing parameters of fibular complication subgroups with the non-complication group. Analysis was performed using the linear mixed-effects model

Fibular complication group	No. of segments	PFM **^a^** p-value	DFM **^b^** p-value	MAD **^c^** p-value
Premature consolidation	10	< 0.001	0.9	0.07
Nonunion	12	0.4	0.02	0.4
Angulation	8	0.8	0.5	0.5

**^a–c^** See Table 1

## Discussion

Distal migration of the proximal fibula during tibial lengthening has been reported in association with distraction osteogenesis (Hatzokos et al. 2004). We found that during a large amount of lengthening, the proximal wire which was inserted through the fibular head and crossed the tibia could not hold the fibula due to high resisting force, and often cut out from the fibular head. This resulted in less distraction of the fibula, premature consolidation of the fibula, and distal migration of the proximal fibula. Our study clearly indicates that cut-out of the wire is related to the lengthening percentage of the fibula. PFM was seen in 120 segments and DFM was seen in 96 segments. However, cut-out of the wire occurred in only 10 segments. This is because even though the transfixed wire did not completely cut out from the proximal fibular cortical end, the wire may migrate proximally in the metaphyseal area of the fibular head or it could be bent due to the resisting force.

Other authors have shown that PFM and DFM occur in spite of tibiofibular fixation due to strong tethering effects of the fibular regenerate and interosseous membrane and due to a disproportionate amount of soft tissue tensioning (Hatzokos et al. 2004, Shyam et al. 2009). Meiss (2000) reported inferior subluxation of the proximal tibiofibular joint. Aldegheri (2000) also agreed that distal migration of the fibular head was a frequent phenomenon in his series of patients, who underwent tibial lengthening without fixation of the proximal segment of the fibula to the tibia. However, he stated that it did not cause instability of the knee. In our study, distal migration of the proximal fibula was associated with a valgus deformity of the knee joint. Even though the patients with PFM have no symptoms, it sometimes requires another surgical procedure to correct the malalignment. One of the patients underwent corrective osteotomy due to valgus deformity, which occurred after lengthening in our study. Valgus mechanical axis deviation was seen in 10 patients and the mean MAD was 8.7 mm of varus at the final follow-up. Although MAD of up to 10 mm is considered to be normal (Paley et al. 1994), it is necessary to keep a close watch on the mechanical axis, as it was found to increase considerably in some patients with substantial distal migration of the proximal tibia. We found that the PFM and MAD increased rapidly in the midrange of 25–50% of lengthening and then slowed down when the lengthening percentage increased to more than 50%. This is similar to the findings of Shyam et al. (2009). Resisting force can hold the fibula and bend the transfixed proximal wire. However, after lengthening of more than 50%, the proximal wire could overcome the resisting force of the attached muscles to some extent.

There was less proximal migration of the distal fibula than distal migration of the proximal fibula, but some of the patients with marked promixal migration of the distal fibula showed valgus tendency of the ankle. Previous publications have described the part played by the distal fibula in the mechanics of the ankle (Lambert 1971, McCullough and Burge 1980, MacNicol and Catto 1982). The lateral talocrural joint is a congruent weight-bearing joint, and thus any change in the length of the fibula leads to mechanical changes characterized by loss of talar rotatory stability and lateral tilting of the talus. This tilt would aggravate the valgus tendency at the ankle joint. In our patients, valgus deformity of the ankle was not seen if the distal fibular length was preserved. Also, the distal wire had a high incidence of tethering of the peroneal muscles, which resulted in heel valgus deformity; thus, careful insertion of the wire is required to avoid this deformity. Another cause of late heel valgus was the proximal migration of the distal fibula, which resulted from the collapse of the fibular lengthening site (Park et al. 2011).

Premature consolidation of the fibular osteotomy site is a troublesome complication that can induce PFM as well as DFM. In our study, premature consolidation occurred in 10 segments even though proximal and distal tibiofibular joints were transfixed with an Ilizarov wire each. Hatzokos et al. (2004) reported 1 case of premature consolidation among 30 tibial segments, even though the proximal part of the fibula was not transfixed to the tibia. The reason for less premature consolidation in Hatzokos’ series was that a segment of fibular bone approximately 1 cm in length was excised to avoid early consolidation. On the other hand, a nonunion rate of 12/30 was reported in that series. In our study, patients with premature consolidation more often showed PFM. This requires repeated osteotomy and additional fixation of proximal and distal tibiofibular joints. Ilizarov wire fixation is often sufficient for stabilization of the syndesmosis during short, uncomplicated lengthening. Also, wire fixation has advantages as it causes little damage to the joint due to small diameter and can be removed by a simple outpatient procedure (Saleh et al. 2002). However, for more high-risk situations such as longer lengthening and premature consolidation of the fibula, the knee and ankle should be protected by additional screws or fibular half-pins.

Nonunion of the fibular osteotomy site was associated with valgus deformity of the tibia and with DFM. This was previously reported in patients with fibular hemimelia, presumably due to absence of lateral rays (Cheng et al. 1998, Epps and Schneider 1989). Ankle valgus deformity can be difficult to treat; thus, even though the fibula is a minor weight-bearing bone, we recommend bone graft and inducing union of the fibula to maintain the alignment of the lower extremities.

Surprisingly, we found that angulation of the fibula itself does not have much effect on malalignment. This is probably because most of our patients were children. We could therefore obtain bone union easily in spite of the angulation of the fibular osteotomy site. If there is no PFM or DFM, angular deformity of the fibula alone does not require surgical intervention or any other special treatment.

In summary, we found an important role of the fibula during tibial lengthening. Insertion of an additional 3- or 4-mm half-pin at the proximal fibula at the initial surgery can be recommended to prevent complications associated with fibular shortening.
